# Non-invasive visual tools for diagnosis of oral cancer and dysplasia: A systematic review

**DOI:** 10.4317/medoral.20996

**Published:** 2016-03-06

**Authors:** Ilaria Giovannacci, Paolo Vescovi, Maddalena Manfredi, Marco Meleti

**Affiliations:** 1 DDS, Msci. Department of Biomedical, Biotechnological and Translational Science-Center of Oral Laser Surgery and Oral Pathology, Dental School, University of Parma, Parma, Italy; 2 DDS, PhD. Department of Biomedical, Biotechnological and Translational Science-Center of Oral Laser Surgery and Oral Pathology, Dental School, University of Parma, Parma, Italy

## Abstract

**Background:**

Gold standard for the diagnosis of oral dysplasia (OD) oral squamous cell carcinoma (OSCC) and malignant lesions is the histological examination. Several adjunctive diagnostic techniques have been proposed in order to increase the sensitivity (SE) and specificity (SP) of conventional oral examination and to improve the diagnostic first level accuracy. The aim of this study is to perform a systematic review on non-invasive tools for diagnosis of OD and early OSCC.

**Material and Methods:**

Medline, Scopus, Web of Knowledge databases were searched, using as entry terms “oral dysplasia AND diagnosis” / ”oral cancer AND diagnosis”. Data extracted from each study included number of lesions evaluated, histopathological diagnosis, SE, SP, positive and negative predictive values (PPV and NPV), diagnostic accuracy (DA) and the main conclusions.

**Results:**

After title and abstract scanning of 11.080 records, we selected 35 articles for full text evaluation. Most evaluated tools were autofluorescence (AF), chemiluminescence (CL), toluidine blu (TL) and chemiluminescence associated with toluidine blue (CLTB).

**Conclusions:**

There is a great inhomogeneity of the reported values and there is no significant evidence of superiority of one tool over the other. Further clinical trials with a higher level of evidence are necessary in order to assess the real usefulness visual diagnostic tools.

**Key words:**Oral dysplasia, oral cancer, diagnosis, visual diagnostic tool, systematic review.

## Introduction

Oral squamous cell carcinoma (OSCC) is the sixth most common malignant tumour, with an incidence of more than 500.000 cases per year (1).

The most important prognostic factor influencing the disease-specific survival rate is the tumour stage at diagnosis. Patients with stage I tumours have a 5-year survival rate of 75%, which dramatically decreases in patients with tumours in stage III or IV, being 49% and 30%, respectively (1,2).

The diagnostic pathway for oral suspicious lesions usually starts with the conventional objective examination (COE) based on inspection and palpation of the oral mucosa with the support of an incandescent light available on the dental chair. It is well known that COE mainly depends on a subjective interpretation, which is a consequence of the experience of the operator. Moreover, oral epithelial dysplasia (OED) and early OSCC may already be present within areas of oral mucosa macroscopically normal, as well as within the context of oral potentially malignant disorders such as leukoplakia, erythroplakia, submucous fibrosis and oral lichen planus (3).

The gold standard for the diagnosis of oral dysplastic and neoplastic malignant lesions is the histological examination (4). Incisional or excisional biopsy techniques are the most reliable methods to collect a surgical specimen suitable for microscopic evaluation. However, despite the little invasivity of such techniques, they still have some disadvantages in terms of morbidity and possible artifacts induced by the method of collection.

In a recent paper, Mehrotra *et al.* indicated that there are two approaches for detection of oral dysplasia and cancer: 1) oral cancer screening programs that identify asymptomatic patients with suspicious lesions and 2) specific diagnostic tools to identify dysplasia and early oral cancers in asymptomatic patients with an oral abnormality (5).

Several visual diagnostic aids have been developed as adjunctive tools in order to increase the diagnostic accuracy (DA) and enhance the specificity (SP) and sensitivity (SE) of the conventional diagnostic pathway. However, results of studies on the usefulness of such tools show impressive discrepancies with regard to values such as the positive or negative predictive values (PPV, NPV), when the same tools is evaluated by different researchers.

The aim of this study is to perform a systematic review on non-invasive tools for the diagnosis of OED and OSCC, taking into account factors as SE, SP, PPV, NPV and DA.

## Material and Methods

The databases Medline, Scopus and Web of Knowledge were searched, using as entry terms “oral dysplasia AND diagnosis” / ”oral cancer AND diagnosis”. No time limits were specified in the present research.

**Figure 1:**
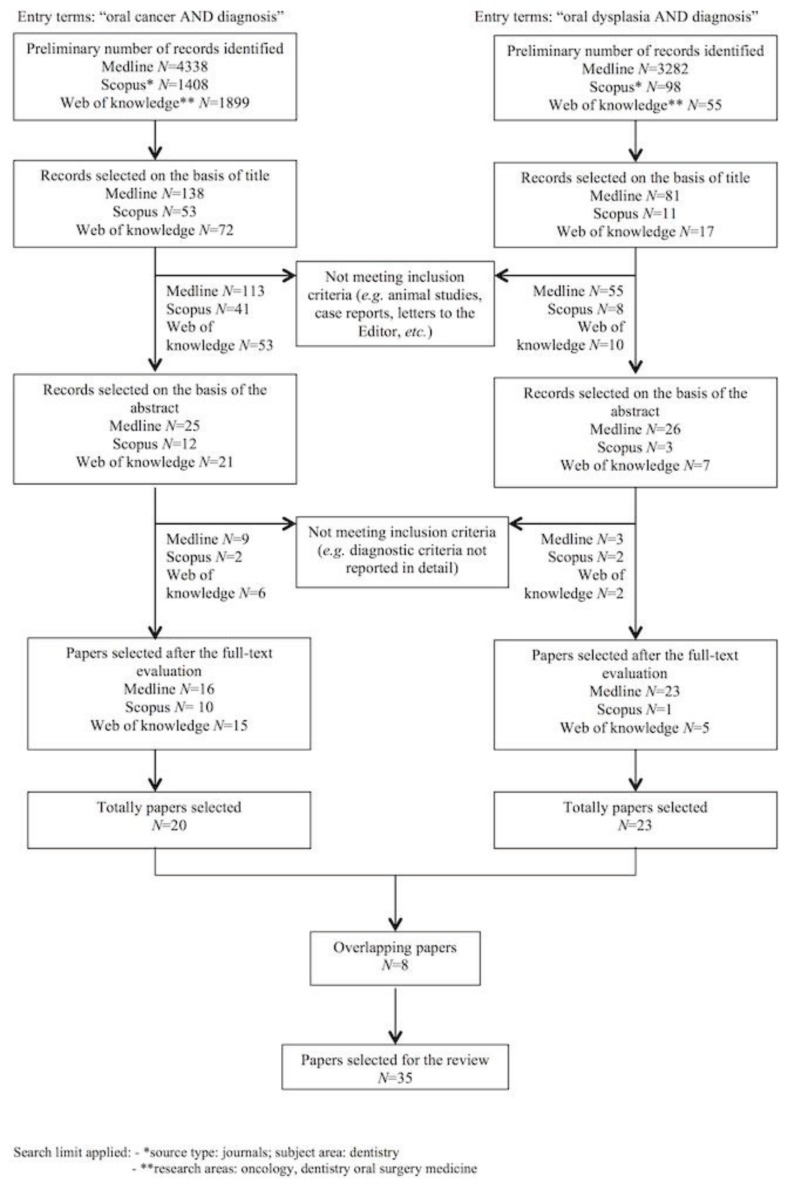
Flow-chart diagram for the selection of the 35 studies included in the present analysis.

Search flow is shown in figure 1. Papers with abstract unavailable were excluded for further evaluation.

Titles and abstract were screened and the following exclusion criteria were applied:

- papers not in English.

- studies *ex vivo* or based on animal models.

- typology of the study: case reports, case series with less than 10 patients, conference proceedings, personal communications, editorials, descriptive studies and reviews.

- studies that analyse COE, invasive diagnostic tools (e.g. scalpel biopsy) or minimally invasive diagnostic tools (e.g. brush biopsy, exfoliative cytology) alone.

- studies that analyse salivary biomarkers.

- studies including also tumours of other head and neck regions (e.g. oropharynx).

Papers with equivocal abstracts were included for full-text evaluation. Further studies were excluded after full-text reading, if not pertinent with aim of the present review.

Data extracted from each study included authors and publication year, typology of the study, diagnostic tool analysed, number of lesions evaluated, (if present) histopathological diagnosis, (if present) SE, SP, PPV, NPV, DA and the main conclusions of the study ( Table 1 and  Table 1Continue, Table 2).

**Table 1:**
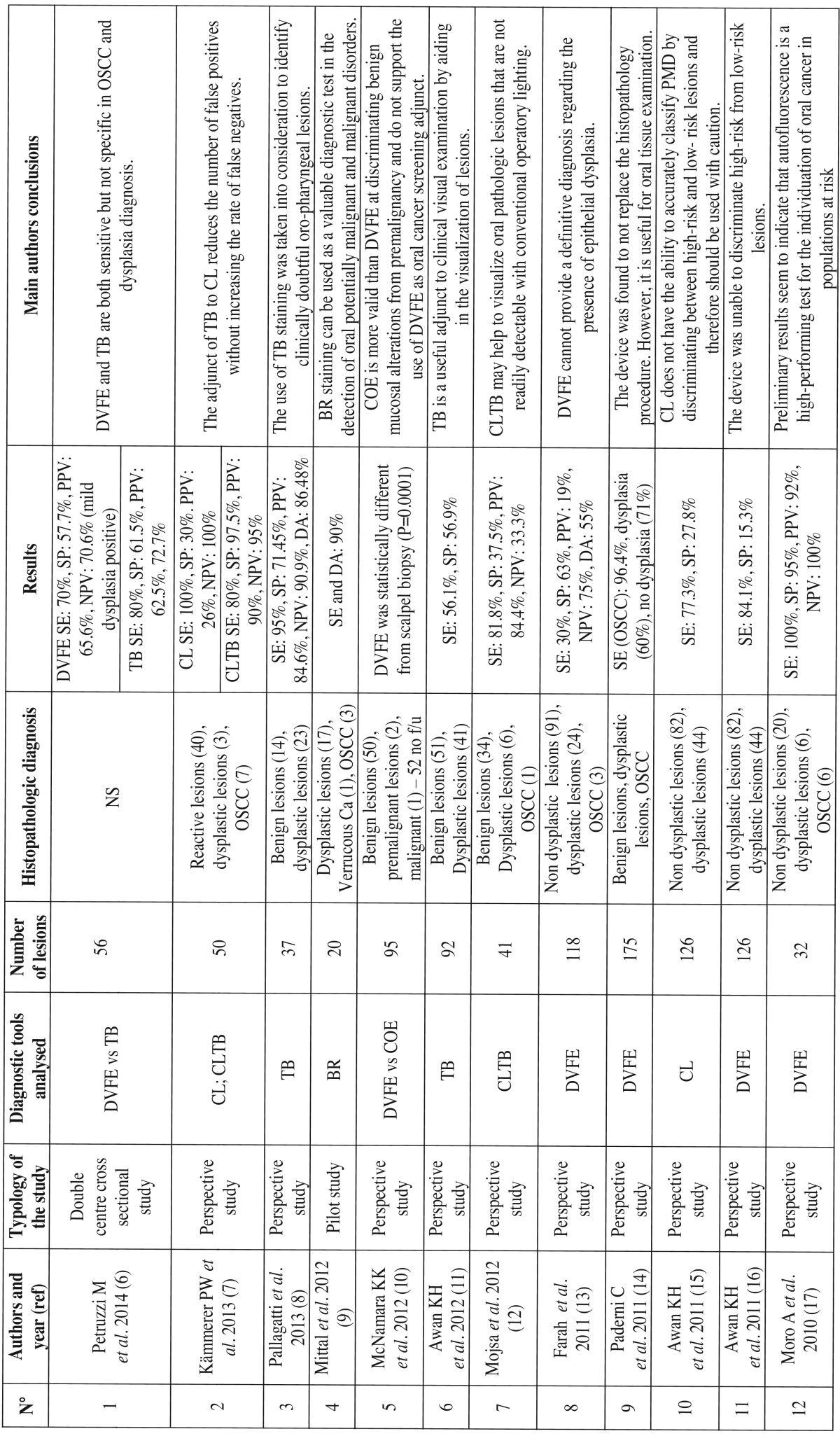
Studies identified using as entry terms “oral dysplasia AND diagnosis”.

**Table 1Continue:**
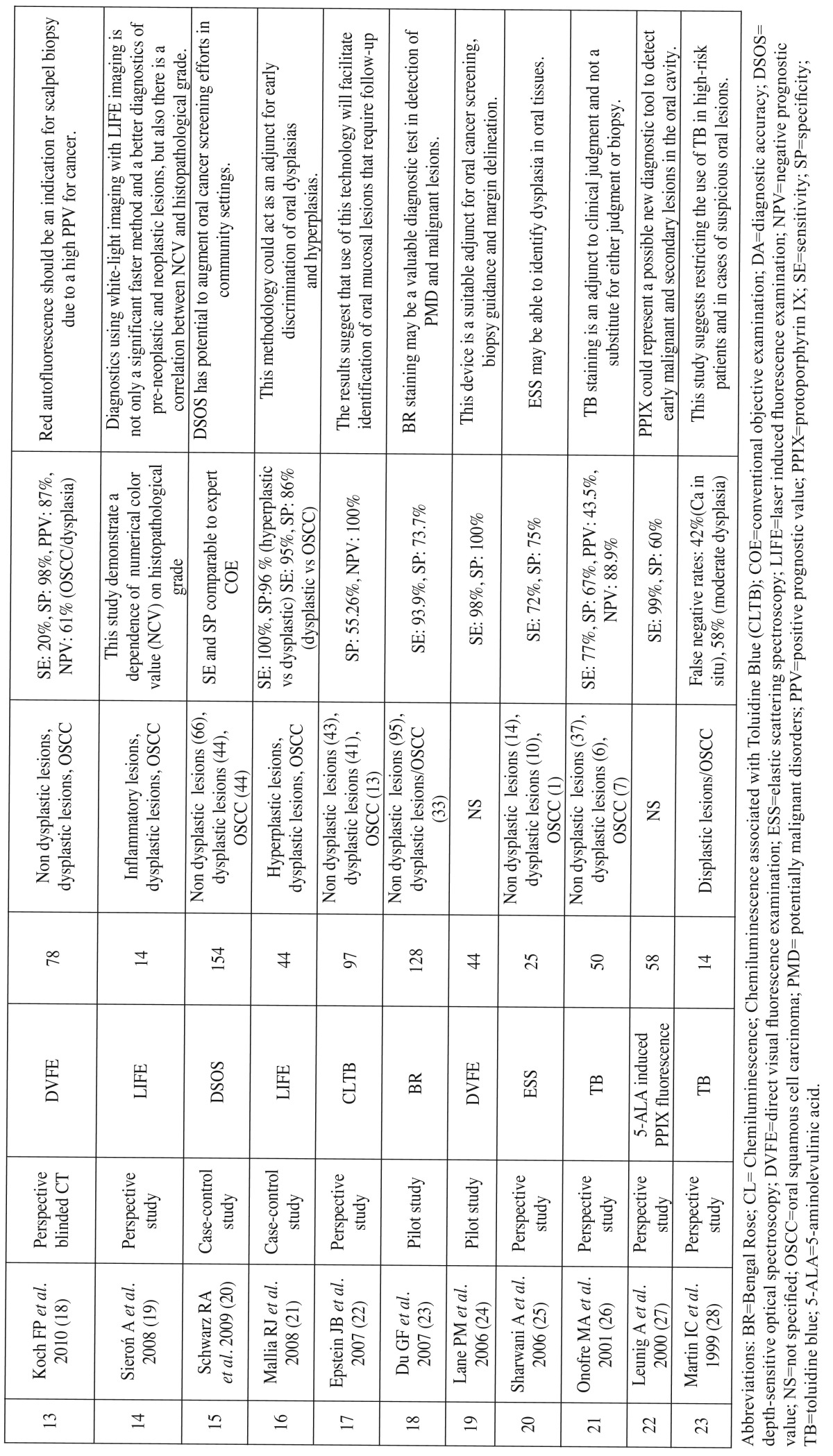
Studies identified using as entry terms “oral dysplasia AND diagnosis”.

**Table 2:**
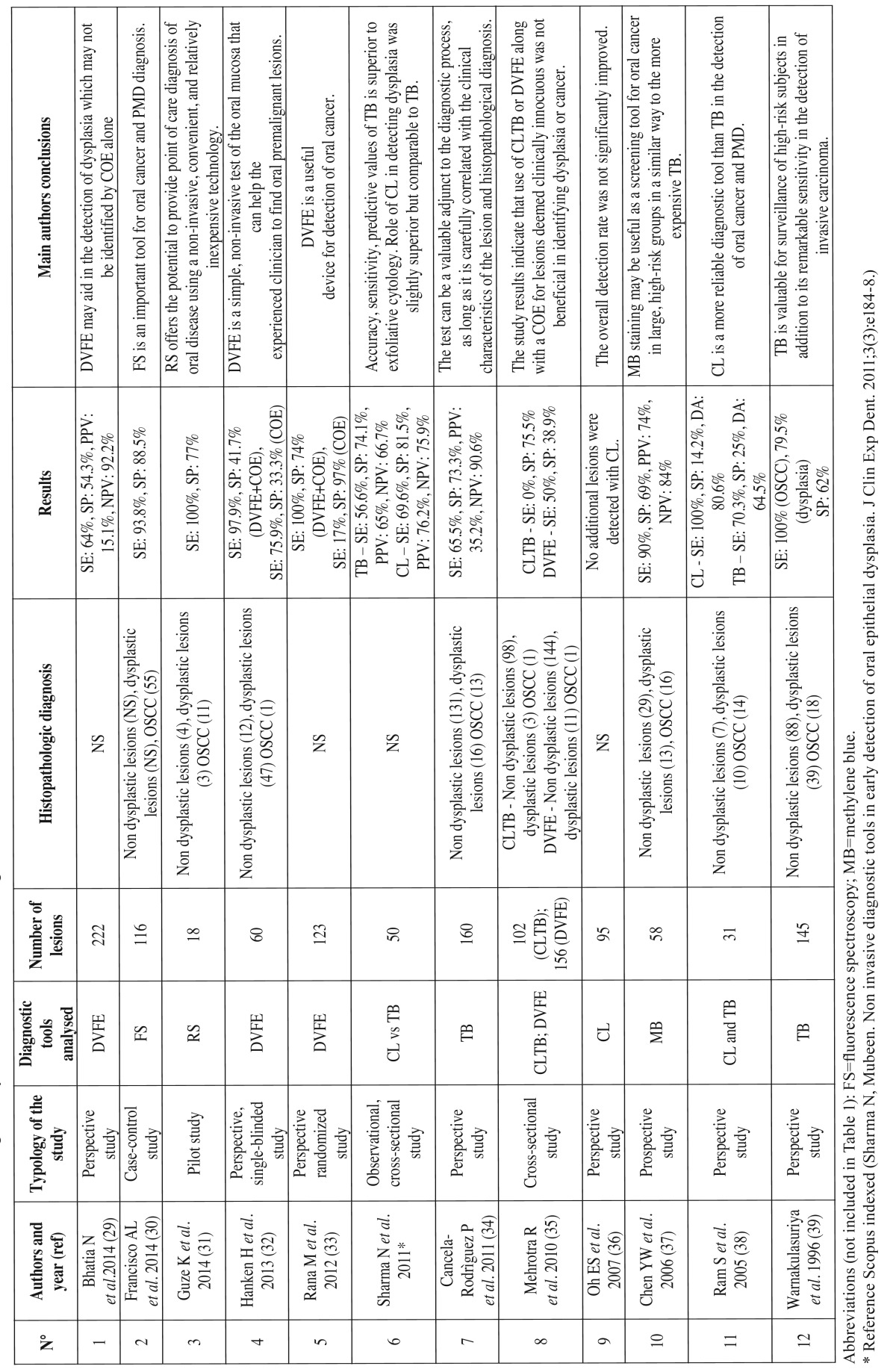
Studies identified using as entry terms “oral cancer AND diagnosis”.

SE and SP measure the accuracy of a test without any relation to the disease or population, whereas PPV and NPV measure the proportion of people whose test results reflect their health status. DA is the proportion of true positive results (both true positive and true negative) in a selected population, with regard to a specific disease.

The mean value of each variable analysed was calculated; range and standard deviation (SD) were indicated for samples having > 2 values.

Level of evidence of each study was assessed according to the Oxford Evidence-based Medicine (OEBM) Levels for Diagnosis updated in March 2009.

## Results

Twenty-three papers were eventually selected for the present systematic review when using “oral dysplasia AND diagnosis” as entry terms. The use of “oral cancer AND diagnosis” as entry terms allowed the identification of further 25 full-text manuscripts (6-39).

Twenty-three studies were perspective (OEBM level: 2b), 4 studies were pilot (OEBM level: 3b), 3 studies were case-control (OEBM level: 4), 4 studies were cross-sectional (OEBM level: 2b). Only one study was a perspective randomized clinical trial (RCT) (OEBM level: 1b).

Eight typologies of non-invasive visual diagnostic tools were identified ( Table 3).

**Table 3:**
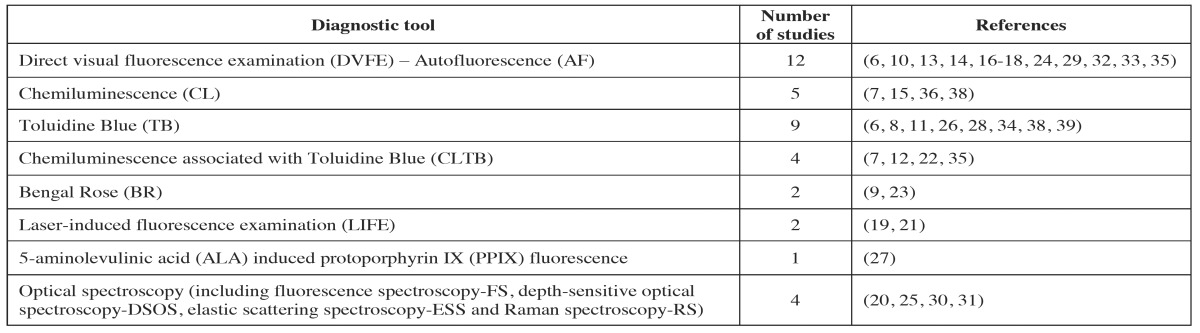
Typology of not invasive visual diagnostic tools identified in this review and number of related studies.

**Figure 2:**
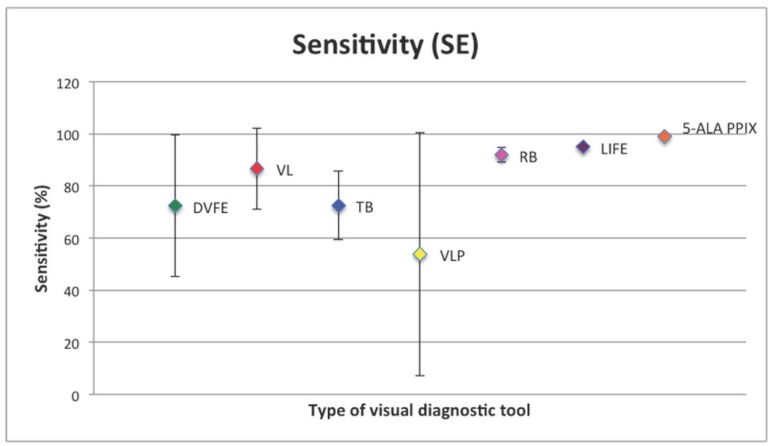
Sensitivity with relative standard deviation of non-invasive visual diagnostic tools analysed. DVFE: Direct visual fluorescence examination. VL: ViziLite®. TB: Toluidine Blue. VLP: ViziLite Plus®. RB: Bengal Rose. LIFE: Laser-induced fluorescence examination. 5-ALA PPIX: 5-aminolevulinic acid (ALA) induced protoporphyrin IX (PPIX) fluorescence.

**Figure 3:**
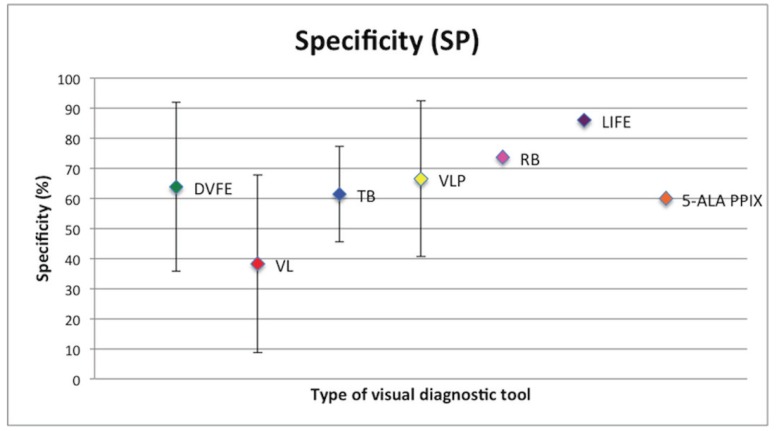
Specificity with relative standard deviation of non-invasive visual diagnostic tools analysed. DVFE: Direct visual fluorescence examination. VL:ViziLite®. TB: Toluidine Blue. VLP: ViziLite Plus®. RB: Bengal Rose. LIFE: Laser-induced fluorescence examination. 5-ALA PPIX: 5-aminolevulinic acid (ALA) induced protoporphyrin IX (PPIX) fluorescence.

Mean SE and SP (with SD) are shown in figures 2,3.

1. Auto fluorescence (AF) - Direct visual fluorescence examination (DVFE) 

Among 12 studies evaluating AF/DVFE, 8 were perspective (OEBM level: 2b), 2 were cross-sectional (OEBM level: 2b), 1 was a pilot study (OEBM level: 3b) and 1 was a perspective RCT (OEBM level: 1b) (6,10,13,14,16-18,24,29,32,33).

Data on SE were reported in 10 studies, while information on SP was available in 11 studies. Mean SE was 72.4% ranging from 20% to 100% (SD = 27.1). Mean SP was 63.79% ranging from 15.3% to 100% (SD = 28.17).

Data on PPV were available in 5 studies (mean: 55.74%, ranging from 15.1% to 92%, SD = 36.71); data on NPV were available in 5 studies (mean: 79.76%, ranging from 61% to 100%, SD = 15.99); DA was reported in 1 study (55%).

2. Chemiluminescence (CL)

Among 5 studies evaluating CL, 4 were perspective (OEBM level: 2b) and 1 was observational cross-sectional (OEBM level: 2b) (7,15,38).

Data on SE and SP were reported in 4 studies. Mean SE was 86.72%, ranging from 69.6% to 100% (SD = 15.65). Mean SP was 38.37%, ranging from 14.2% to 81.5% (SD = 29.59).

Data on PPV and NPV were available in 2 studies (mean PPV: 74.5%; mean NPV: 63%); DA was reported in 1 study (80.6%).

3. Toluidine Blue (TB)

Among 9 studies evaluating TB, 7 were perspective (OEBM level: 2b) and 2 were cross-sectional (1 perspective cross-sectional and 1 observational cross-sectional) (OEBM level: 2b) (6,8,11,26,28,34,38,39).

Data on SE and SP were available in 8 studies. Mean SE resulted 72.5%, ranging from 56.1% to 95% (SD = 13.13). Mean SP resulted 61.4%, ranging from 25% to 74.1% (SD=15.95).

Data on PPV were available in 5 studies (mean: 58.16%, ranging from 35.2% to 84.6%, SD=19.4); data on NPV were available in 5 studies (mean: 95.3%, ranging from 66.7% to 90.9%, SD=11.42); data on DA were available in 2 studies (mean: 75.49%).

A perspective study evaluating Methylene Blue (MB) was also identified. In this study SE (90%), SP (69%), PPV (74%) and NPV (84%) were available (37).

4. Chemiluminescence associated with Toluidine Blue (CLTB)

Among 4 studies evaluating CLTB, 3 were perspective (OEBM level: 2b) and 1 was cross-sectional (OEBM level: 2b) (7,12,22,35).

Data on SE were available in 3 studies, while data on SP were available in 4 studies. Mean SE was 53.93%, ranging from 0% to 81.8% (SD = 46.72). Mean SP was 66.44%, ranging from 37.5% to 97.5% (SD=25.88).

Data on PPV were available in 2 studies (mean: 87.2%); data on NPV was available in 3 studies (mean: 76.1%, ranging from 33.3% to 100%). DA was not reported in any study.

5. Bengal Rose (BR)

The 2 studies evaluating BR were pilot studies (OEBM level: 3b) (9,23).

Data on SE were available in both the papers (mean: 91.95%); mean SP was available in 1 study (73.7%).

Data on PPV and NPV were not available in any study, while DA was reported in 1 study (DA: 90%).

6. Laser-induced fluorescence examination (LIFE)

Among the 2 studies evaluating LIFE, 1 was perspective and 1 was a case-control study (OEBM level: 2b and 4, respectively) (19,21).

Data on SE and SP were available in 1 study. SE ranged from 100% to 95% and SP ranged from 96% to 86% taking into account the histopathological diagnosis.

Data on PPV, NPV and DA were not reported in any study.

7. 5-aminolevulinic acid (ALA) induced protoporphyrin IX (PPIX) fluorescence

Only one perspective study evaluating this tool was included in the present research (OEBM level: 2b) (27).

SE was 99%; SP was 60%. Data on PPV, NPV and DA were not available.

8. Optical spectroscopy

One case-control study regarding fluorescence spectroscopy (FS), one pilot study regarding Raman spectroscopy (RS), one perspective study regarding Elastic scattering spectroscopy (ESS) and one case-control study regarding an experimental assessment of depth-sensitive optical spectroscopy (DSOS) were identified (OEBM level: 4, 3b, 2b and 4, respectively) (20,25,30,31).

Among these, SE and SP were available for FS (SE: 93.8%, SP: 88.5%), RS (SE: 100%, SP: 77%) and ESS (SE: 72%, SP: 75%) (25,30,31).

Data on PPV, NPV and DA were not available in any study.

## Discussion

The principles of functioning of non-invasive visual diagnostic tools for OSCC and dysplastic lesions are very different, being based on diverse specific cellular and tissue characteristics. Such a great diversity may partly explain the impressive discrepancy of results obtained in the studies analysed. Another reason which can give some reasons for the wide range of results, in terms of SE, SP and DA is the great variability both of the typology of the studied lesions and of the diagnostic criteria used for the clinical and histological assessment of such lesions. The difficulty to establish univocal and broadly-accepted criteria for the assessment of the OED has been widely reported, particularly, with regard to the inter- and intra-observer disagreement for the diagnosis.

Moreover, SE and SP may well depend on the degree of development of a lesion, seeming quite reasonable that both these indicators increase with the progression of a lesion from normal, to dysplastic, early neoplastic and invasive and destructive lesion.

Taking into account the above mentioned considerations, we report a short discussion for each tool analysed:

- Auto fluorescence (AF) - Direct visual fluorescence examination (DVFE)

Auto fluorescence (AF) uses natural fluochromes which are located within the epithelium and the submucosa and which are excited when irradiated with specific wavelengths. Using wavelengths between 375 and 440 nm, some fluochromes show fluorescence in the range of the green colour. Following such irradiation, normal, unaltered mucosa emits a pale green AF light when viewed through a selective, narrow-band filter. A proper filtration is crucial, due to the intense light used for excitation of the fluorochromes (13,15). Areas of reduced AF (dark areas) are suspicious for epithelial dysplasia or OSCC, whereas normal mucosa appears bright green (10).

The VELscopeTM (LED Medical Diagnostics Inc., Barnaby, Canada) system consists of a non-invasive device designed to visualise early mucosal changes using the principles of tissue AF. According to such principles, dysplastic changes should be associated with a loss of stromal AF (29,32). It seems of paramount importance to highlight here that benign lesions, or those associated to inflammation, can also be characterized by a loss of stromal AF, which grossly limits the diagnostic specificity, especially in low-risk populations.

Mean SE and SP for this tool, were 72.4% and 63.79%, respectively. It is opinion of the authors that such values, at the moment, are unacceptable for a tool specifically dedicated to the diagnosis of oral mucosal malignant lesions. However, it should be stressed that there are apparently no other non-invasive visual diagnostic tools significantly better than AF-based tools.

It is somewhat surprising that values of SE range from 20% to 100% and value of SP goes from 15.3% to 100%.

Level of EBM for the selected studies seem to be quite acceptable, being ≥ 2b for all the studies, except one (3b level) (24). It is worthy mentioning that the study with the highest EBM level (1b) showed high values both of SE and SP (100% and 74%, respectively) (33).

- Chemiluminescence (CL)

The ViziLite® (VL - Zila Pharmaceuticals, Phoenix, AZ) was the first FDA-approved (2002) adjunctive technology to conventional head and neck examination for improving visualization of early dysplastic or neoplastic lesions. This system involves an oral rinse with a 1% acetic acid solution for 1 minute, to remove the glycoprotein barrier and slightly desiccate the oral mucosa. A diffuse chemiluminescent blue/white light with an average wavelength of 490 to 510 nm is then activated and used to examine the oral tissues. Normal cells absorb the light and appear blue, whereas abnormal cells have a higher nuclear/cytoplasmic ratio and should reflect the light appearing whiter with brighter, sharper, more distinct margins (15,36,38).

Mean SE and SP resulted 86.72% and 38.37%, respectively. All the analysed studies have an EBM level of 2b, but there is a great inhomogeneity especially for SP, which ranges from 14.2% to 81.5%.

- Toluidine Blue (TB)

Toluidine blue (TB), also known by its chemical name tolunium chloride (TC), is a cationic met achromatic dye that may selectively bind to free anionic groups such as sulfate, phosphate, and carboxyl ate radicals of large molecules. It has been used for decades as aid to the identification of mucosal abnormalities of the cervix as well as those in the oral cavity (8).

TB stains deoxyribonucleic acid and/or may be retained in intracellular spaces of dysplastic epithelium, which clinically appears as royal blue areas. It is postulated that the increased amount of DNA and RNA in neoplastic cells and the wider intercellular canals compared to normal epithelial cells are responsible for staining malignant cells (11).

Mean SE and SP were 72.5% and 61.4%, respectively. These values are poorly acceptable in oncologic diagnosis and they seem to be more realistic because standard deviations are lower than those calculated for the other diagnostic tools.

- Chemiluminescence associated with Toluidine Blue (CLTB)

In order to reduce the high number of false positive cases obtained through VL, the manufacturer added TB (ViziLite Plus® - VLP) (12,22).

Data related to the use of this technique are very poor and discordant; mean SE and SP were 53.93% and 66.44%, respectively, but standard deviations were excessively high.

- Rose Bengal (RB)

Rose Bengal (RB) is the 4,5,6,7-tetrachloro- 2’,4’,5’,7’-tetraiodo-derivative of fluorescein. It has been widely used to diagnose various ocular surface disorders. It has been believed to stain desquamated ocular epithelial cells, dead or degenerated cells but not healthy epithelial cells. RB staining was even used to delineate the extent of corneal and conjunctival neoplasms. Therefore, such findings of RB enlightened us to carry out researches in detection of oral precancerous and malignant lesions (9,23).

Data on SE and SP related to this tool are scarce and resulting from studies of low OEBM level (3b).

- Laser-induced fluorescence examination (LIFE)

This technique is based on AF of the tissue as well as DVFE. The instrumentation proposed by Mallia *et al.* is comprised of a diode laser (Stocker Yale, Canada, 404 nm, 50 mW, CW) for excitation of tissue fluorophores (21). Light emission from the laser source is guided to the oral mucosa through a 3 µm long bifurcated fiber optic probe that has a central fiber to deliver the excitation beam and 6 surrounding fibers (400 µm diameter each) to collect AF emissions. The red to green colour ratio is defined as the numerical color value (NCV).

Two studies regarding this tool have been selected for this review. SE and SP values are reported in 1 study only and they are high (SE: 100%-95%; SP: 96%-86%, according to the histopathological diagnosis), but the OEBM level is low (4). Data from further studies with a higher OEBM level are necessary.

- 5-aminolevulinic acid (ALA) induced protoporphyrin IX (PPIX) fluorescence

Only one perspective study describing this technique was selected for this review (29). Topical or systemic administration of 5-ALA results in a selective accumulation of PPIX in neoplastic tissue, which is probably due to altered activity levels of the enzymes of the heme biosynthetic pathway within malignant transformed cells. In the protocol of Leuing *et al.,* the patients performed a 15-minute continuous rinsing of the oral cavity using the 5-ALA solution. After an incubation period of 1 to 2.5 hours (maximum contrast after 1.5h), fluorescence investigation was performed. In 13.8% of the patients, additional findings like dysplasia, carcinoma in situ, OSCC were found through fluorescence in contrast to COE (27). An evaluation of the biopsy specimens resulted in a SP of 60% and a SE of 99% (29). ALA-induced fluorescence could represent a possible useful new diagnostic tool to detect early malignant lesions in the oral cavity. However, further studies seem to be necessary.

- Optical spectroscopy

Optical spectroscopy is a non-invasive diagnostic method that has been investigated in many forms including fluorescence spectroscopy (FS), elastic or diffuse scattering spectroscopy (ESS), and Raman spectroscopy (RS). Spectroscopic measurements can detect biochemical and architectural alterations in tissue that are related to the carcinogenesis. These alterations may include changes in the concentrations of native fluorophores such as collagen, elastin, keratin, nicotinamide adenine dinucleotide (NADH), and flavin adenine dinucleotide (FAD); changes in hemoglobin concentration and oxygenation; increasing epithelial thickness; increasing nuclear size and nuclear/cytoplasmic ratio; change in vascularization (20,25,30,31).

These principles are employed within experimental methods; SE and SP values seem to be high, but there is need for more data. Only one study for each optical spectroscopy method was identified and they had a low OEBM level (4, 3b and 2b, respectively).
